# Resolution enhancement on single-shot X-ray spectrometers using a detuned non-dispersive multi-crystal analyzer

**DOI:** 10.1107/S1600577525000505

**Published:** 2025-02-17

**Authors:** Taito Osaka, Yuichi Inubushi, Takashi Kameshima, Ichiro Inoue, Makina Yabashi

**Affiliations:** a RIKEN SPring-8 Center, 1-1-1 Kouto, Sayo-cho, Sayo-gun, Hyogo679-5148, Japan; b Japan Synchrotron Radiation Research Institute 1-1-1 Kouto, Sayo-cho Sayo-gun Hyogo679-5198 Japan; SLAC National Accelerator Laboratory, USA

**Keywords:** self-amplified spontaneous emission, X-ray free-electron lasers, dynamical diffraction, single-shot X-ray spectrometers, point spread functions, angular spread functions, multi-crystal analyzers

## Abstract

This study proposes and demonstrates a simple method for improving the energy resolution in a single-shot X-ray spectrometer, which consists of a focusing mirror and a single-crystal analyzer. Two Si(220) channel-cut crystals arranged in a non-dispersive geometry are employed as the analyzer. The angular width of diffraction for the multi-crystal analyzer is reduced by detuning one of the crystals, thereby enhancing the energy resolution of the spectrometer while maintaining the energy range. A proof-of-principle experiment with 10.4 keV X-rays clearly shows a resolution enhancement by a factor of two. It was found that X-ray penetration within the crystals broadened the point-spread function on the detector, significantly impacting the energy resolution under highly detuned conditions. A long detector distance of greater than 14 m is expected to achieve a high energy resolution of 100 meV and a range of 80 eV, enabling full spectral characterization of X-ray free-electron laser radiation as well as advanced spectroscopy techniques.

## Abstract 2

Resolution enhancement on a single-shot X-ray spectrometer with a detuned non-dispersive multi-crystal analyzer is proposed and demonstrated, indicating the promising potential for capturing the full spectral information, including the fine-spike structure in self-amplified spontaneous emission X-ray free-electron laser radiation.

## 1. Introduction

Measuring single-shot X-ray spectra is a key approach to characterize both spectral and temporal properties of individual X-ray free-electron laser (XFEL) pulses generated through the self-amplified spontaneous emission (SASE) principle (Huang & Kim, 2007 ▸). Spectra of SASE–XFEL pulses involve numerous narrow spikes over a wide bandwidth and their structure fluctuates shot-by-shot due to the stochastic nature of initial amplification processes in SASE. Through the Fourier-transform relationship between frequency and time, the width of each spike of typically several hundred millielectronvolts and the overall bandwidth of a few tens of electronvolts can be connected to the pulse duration and coherence time of the XFEL pulse (Yabashi *et al.*, 2006 ▸; Inubushi *et al.*, 2012 ▸), respectively, which are essential characteristics for most measurement methods with XFELs.

A dispersive X-ray spectrometer, in which angular dispersion is introduced to an XFEL pulse and its spectral information is encoded in space, is a powerful tool for capturing single-shot XFEL spectra with a high energy resolution. Mainly two types of dispersive spectrometers have been routinely operated at XFEL facilities. One of them employs a bent-crystal analyzer (Zhu *et al.*, 2012 ▸; Makita *et al.*, 2015 ▸; Kujala *et al.*, 2020 ▸) and the other a flat-crystal analyzer following a focusing mirror (Yabashi *et al.*, 2006 ▸; Inubushi *et al.*, 2012 ▸). For both types of spectrometers, there is a trade-off relationship between the energy resolution and range. The resolution is, for most cases, mainly attributed to the angular width of Bragg diffraction for monochromatic X-rays. On the other hand, the energy range is determined by the range of the incidence angle on the analyzer crystal. These parameters are closely connected to each other; the use of high-order diffraction, in general, achieves a high resolution with a narrow range. To evaluate detailed spectra of SASE–XFEL pulses, it is necessary to achieve an energy resolution of ∼100 meV and a range of >50 eV over a wide operational photon energy range around 10 keV.

Inubushi *et al.* (2017 ▸) have succeeded in the measurement of full spectra for SASE–XFEL pulses at 9 keV, using an Si(553) flat-crystal analyzer and a multilayer-coated focusing mirror, which generates a highly divergent XFEL beam with an angular divergence of 22 mrad (Matsuyama *et al.*, 2018 ▸) that is 5–10 times wider than that for conventional total-reflection mirrors. They achieved an energy resolution of ∼80 meV and a range of 55 eV. The operational photon energy is, however, strictly limited by the multilayer mirror. Note that a theoretical study suggested that a bent-crystal spectrometer with optimized parameters can meet both the resolution and the range requirements (Kaganer *et al.*, 2021 ▸), though no experimental demonstration has been reported.

In this paper, we propose and experimentally demonstrate a simple method for improving the energy resolution beyond the limitation from the intrinsic Darwin width of Bragg diffraction while maintaining the energy range. We employed a detuned multi-crystal analyzer (MCA) arranged in a non-dispersive geometry with a conventional focusing mirror and successfully made the energy resolution approximately half of that for a single flat-crystal analyzer. We also found that the broadening of the point spread function (PSF) due to X-ray penetration within the analyzer crystals significantly impacted the energy resolution under highly detuned conditions. The detailed concept, experimental results and discussion for future improvements of the dispersive spectrometer are presented.

## 2. Spectrometer concept

First, we briefly introduce the energy resolution δɛ and range Δ*E* for the flat-crystal spectrometer. These parameters are often described by (Inubushi *et al.*, 2012 ▸)
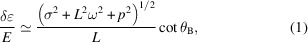

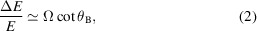
where *E* is the photon energy, σ is the source size, *L* is the source-to-detector distance, ω is the angular width of Bragg diffraction (in other words, the angular resolution of the analyzer), θ_B_ is the Bragg angle at photon energy *E* and Ω is the angular divergence of the incident X-ray beam. *p* is the width of the PSF, which can be rewritten as *p* = 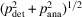, where *p*_det_ and *p*_ana_ represent the detector and the analyzer, respectively. The details of *p*_ana_ will be discussed later. A dominant factor for resolution is ω which is typically defined by the intrinsic Darwin width of Bragg diffraction for a single flat-crystal analyzer (ω_single_). On the other hand, the energy range is mainly determined by Ω. Note that both parameters are proportional to cotθ_B_ which is defined by the diffraction plane of the analyzer and the photon energy (or wavelength) through the well known Bragg equation
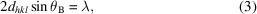
where *d*_*hkl*_ is the lattice spacing of the diffraction plane (*hkl*) and λ is the wavelength of X-rays. In general, a high energy resolution can be achieved using high-order diffraction with a small *d*_*hkl*_ (and a narrow ω_single_), whereas the energy range is made narrow because of a small cotθ_B_ in comparison with a low-order diffraction with a large *d*_*hkl*_. Consequently, there is a trade-off relationship between the energy resolution and range.

**Figure 1:**
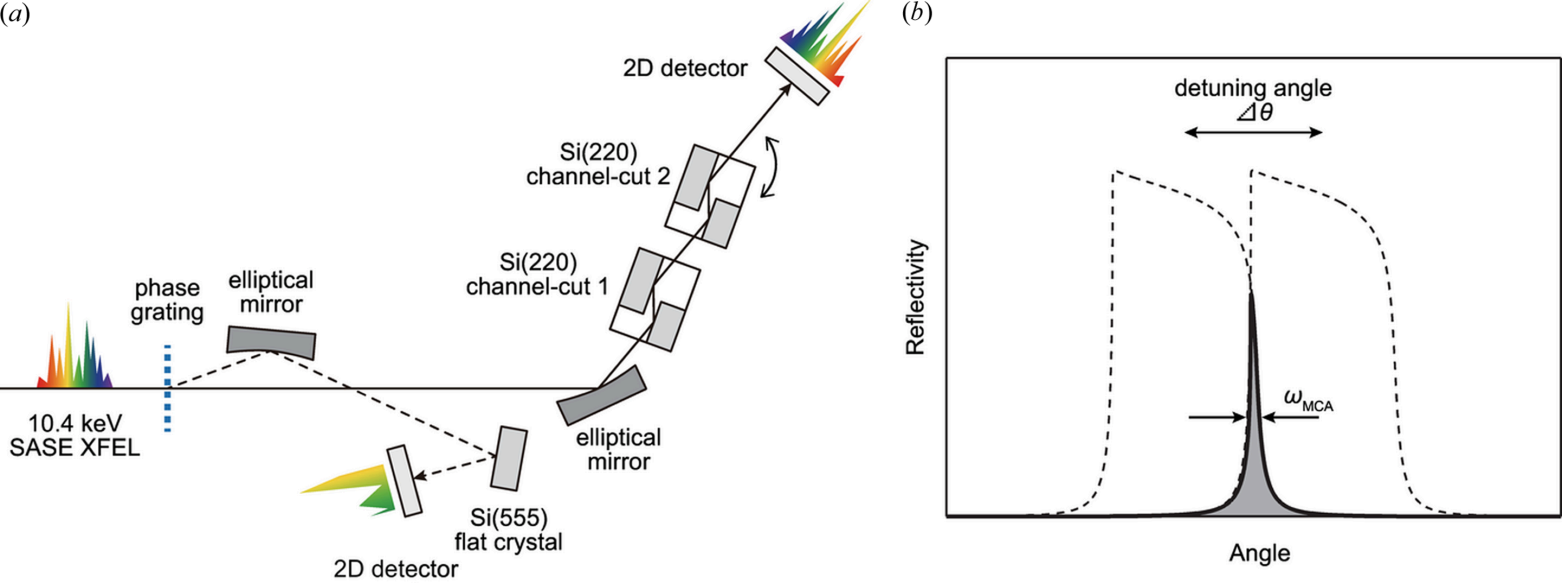
(*a*) Schematic layout of the experimental setup; see text for details. (*b*) Conceptual illustration of an angular resolution function of a multi-crystal analyzer. Dashed lines are the intrinsic Darwin curves of individual crystals with an angular offset Δθ. Filled plot shows the product of the two Darwin curves corresponding to the angular resolution function of the whole analyzer.

The idea of the resolution enhancement for the dispersive spectrometer is the same as the so-called three-crystal configuration proposed by DuMond (1937 ▸) for crystal characterization with high angular sensitivity. In this configuration, the lattice perfection of a sample crystal is characterized with a set of perfect collimator and analyzer crystals arranged in a non-dispersive geometry. Unlike a typical two-crystal configuration, the angular resolution can be enhanced beyond the intrinsic Darwin width by slightly detuning one of the collimator/analyzer crystals because the angular resolution function of the system is defined as the product of the two Darwin curves with an angular offset [Fig. 1 ▸(*b*)]. On the same basis, for the dispersive spectrometer, only the angular resolution ω can be made narrower while maintaining cotθ_B_. By accurately detuning one of the analyzer crystals, the angular resolution of the MCA can be reduced by a factor of ∼10 from the intrinsic Darwin width. Note that similar detuning methods have been routinely utilized in X-ray crystal monochromators at synchrotron facilities to reject higher-order harmonics as well as to reduce the bandwidth (Hart & Rodrigues, 1978 ▸; Mills & Pollock, 1980 ▸; Bonse *et al.*, 1983 ▸) and in measurements of small-angle X-ray scattering properties of a sample material (Bonse & Hart, 1966 ▸; Ishikawa *et al.*, 1985 ▸).

## 3. Experimental

We performed a proof-of-principle experiment at beamline 3 (BL3; Tono *et al.*, 2013 ▸) of the SPring-8 Angstrom Compact free-electron LAser (SACLA) in Japan (Ishikawa *et al.*, 2012 ▸). Fig. 1 ▸(*a*) illustrates the schematic layout of the experimental setup. A 10.4 keV XFEL beam, with a divergence of a few microradians, is first focused in the dispersion plane with a total-reflection elliptical focusing mirror. The divergent beam from the secondary source at the focus is then reflected multiple times by an MCA. As the incidence angle to the analyzer crystal varies spatially along the dispersion direction (vertical here), the spatial profile of the reflected beam provides spectral information about the incidence beam according to equation (3). In this setup, we achieved an angular divergence of Ω ≃ 2.8 mrad and a small σ of ∼200 nm full width at half-maximum (FWHM) (Inubushi *et al.*, 2012 ▸). The reflected beam profile is recorded with a 2D detector at *L* ≃ 2.3 m.

Two Si(220) channel-cut crystals were used as the MCA, with each analyzer crystal reflecting the X-ray beam twice (a total of four reflections). The channel-cut crystals offer two main advantages: (1) they suppress the tail in the angular resolution function of each analyzer crystal through double-bounce reflections; and (2) they maintain the optical axis of the reflected beam parallel to the original axis, even under detuned conditions, due to the intrinsically parallel lattice planes in the crystal blades formed within a monolithic crystal block. The second characteristic also makes the spectrometer less sensitive to angular fluctuations of the incident beam and/or angular vibrations of the analyzer. The channel-cut crystals used in this experiment were polished with a plasma-etching technique (Mori *et al.*, 2000 ▸) to remove damaged layers on the inner-wall reflecting surfaces, resulting in nearly speckle-free reflected X-rays (Hirano *et al.*, 2016 ▸; Matsumura *et al.*, 2024 ▸).

The energy range and resolution for the single-analyzer setup are estimated to be 87 eV and 590 meV, respectively, with *p* = 0. For reference, we also employed a conventional spectrometer with a single Si(555) flat-crystal analyzer (Katayama *et al.*, 2016 ▸), which captures XFEL spectra replicated by a phase-grating splitter. This reference spectrometer had an energy resolution and range of 58 meV (64 meV pixel^−1^) and ∼7 eV, respectively.

A key technical challenge for this scheme is achieving both high angular resolution (*p*_det_/*L* < 2 µrad) and wide coverage (>2.8 mrad) on the 2D detector simultaneously. To meet these requirements, we used a photodiffusion-free transparent scintillator detector (DIFRAS), which consists of a 5 µm-thick Ce-doped Lu_3_Al_5_O_12_ (LuAG:Ce) scintillator bonded to a non-doped LuAG substrate (Kameshima *et al.*, 2019 ▸), along with a large format microscopic system (Kameshima & Hatsui, 2022 ▸). This system was equipped with a distortion-free 7× objective lens system and a CMOS sensor with 14192 × 10640 pixels (Sony, IMX411). Only a portion of full images (14192 × 1500 pixels) was recorded to reduce data size and increase the frame rate from 1 Hz to 6 Hz. The detector field of view was ∼7.6 mm in the dispersion direction, with an effective pixel size of 0.54 µm × 0.54 µm, providing an angular coverage of ∼3.2 mrad and a detector resolution of *p*_det_/*L* < 0.43 µrad with *p*_det_ < 1 µm FWHM. This high spatial/angular resolution of the detector minimized its impact on the energy resolution, enabling detailed characterization of the effect of the detuned MCA.

**Figure 2:**
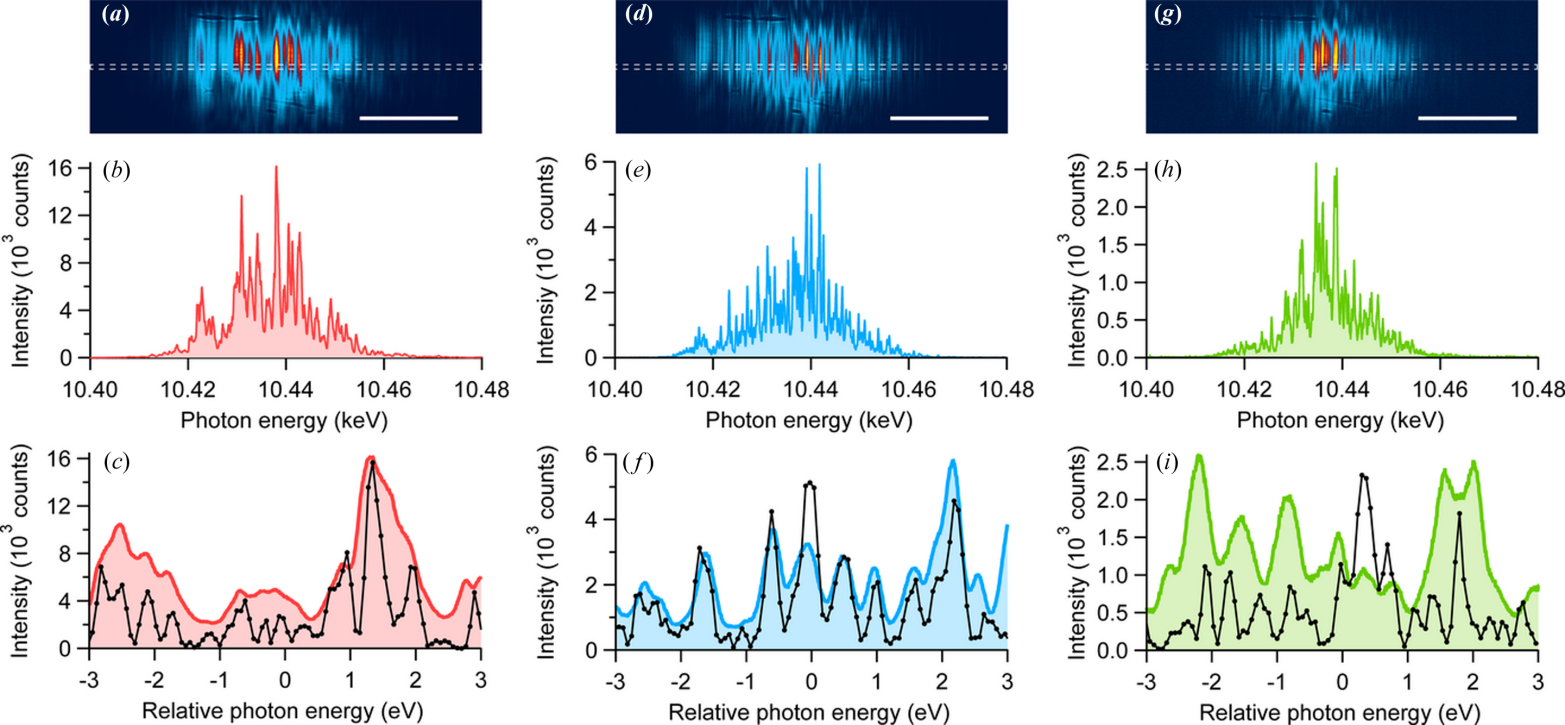
Examples of measured spectra at (*a*)–(*c*) Δθ = 0 µrad, (*d*)–(*f*) 13 µrad and (*g*)–(*i*) 17 µrad. The top row (*a*), (*d*) and (*g*) shows spatial profiles at the 2D detector where the horizontal axis corresponds to the dispersion direction. Scale bar denotes 20 eV. The middle row (*b*), (*e*) and (*h*) presents line profiles averaged over the dashed rectangular areas in the top row. The bottom row (*c*), (*f*) and (*i*) is their magnified spectra. Filled plots are measured with the MCA and black plots represent the reference spectra.

Fig. 2 ▸ shows example spectra measured at detuning angles Δθ of 0 µrad [Figs. 2 ▸(*a*)–2 ▸(*c*)], 13 µrad [Figs. 2 ▸(*d*)–2 ▸(*f*)] and 17 µrad [Figs. 2 ▸(*g*)–2 ▸(*i*)]. Note that the effective energy range was limited in the central ∼50 eV, as the reflected beam profile near the edge was significantly distorted by Fresnel diffraction from the finite mirror aperture of ∼230 µm, which was smaller than the beam size of ∼400 µm FWHM. At Δθ = 0 µrad the fine spike structure observed in the reference spectrum [black line in Fig. 2 ▸(*c*)] appeared significantly blurred, with no distinct spikes visible due to insufficient energy resolution. In contrast, at Δθ = 13 µrad the measured spectra contained fine spikes that matched the reference in both number and location [see Fig. 2 ▸(*f*)]. Further increasing Δθ broadened the spike structure and disrupted the correlation between the measured and reference spectra [see Fig. 2 ▸(*i*)].

**Figure 3:**
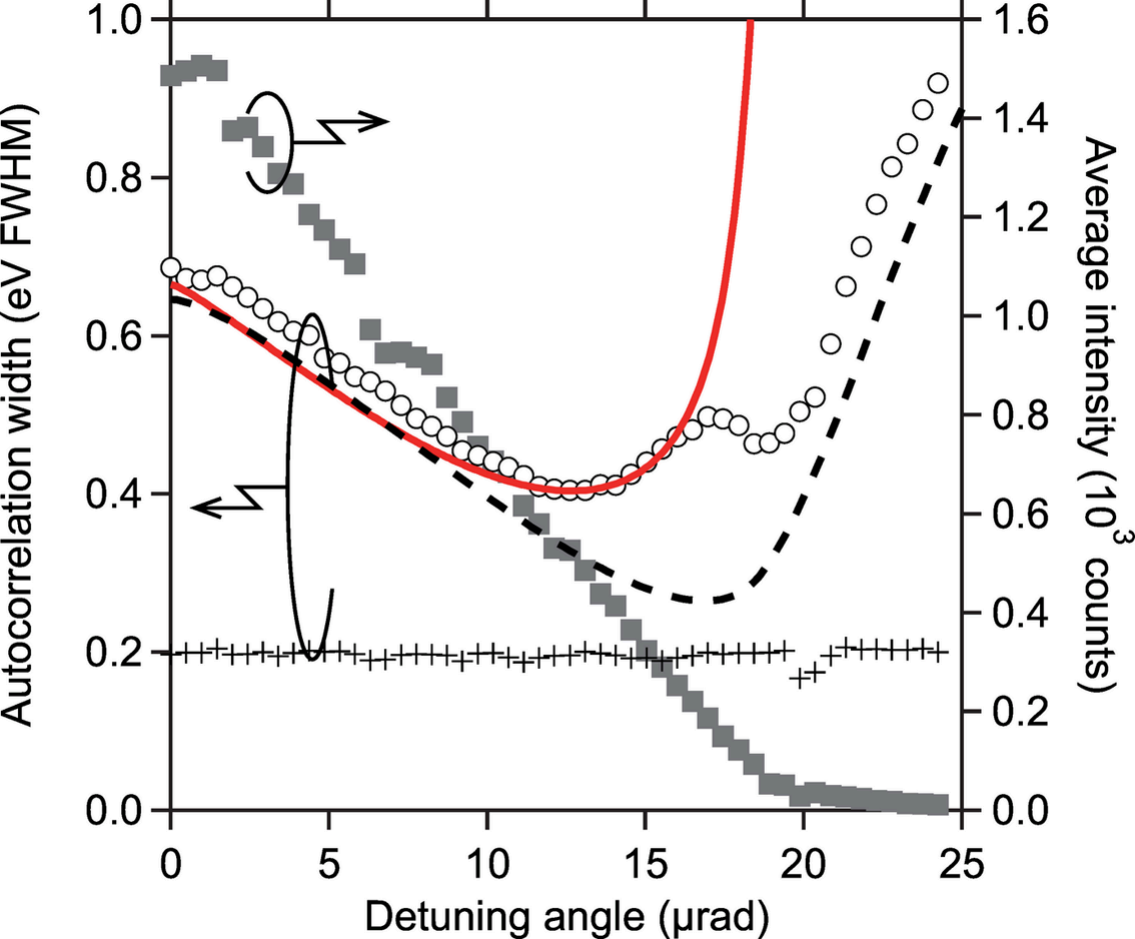
Averaged autocorrelation width of measured (circles) and reference (crosses) spectra (left axis). Solid and dashed lines represent the autocorrelation width of calculated angular resolution functions of the MCA with and without influence of the X-ray penetration within the analyzer crystals, respectively. Both calculated curves are convolved with the SASE spike expected from the reference data. The rectanglular plot represents the averaged intensity (right axis) that corresponds to the rocking curve of the second analyzer crystal.

To evaluate the energy resolution quantitatively, autocorrelation functions of the measured spectra were calculated. Fig. 3 ▸ represents the width of the autocorrelation functions, averaged over 500 shots at each Δθ. The autocorrelation width of spectra measured with the MCA (circles) decreased as Δθ increased up to ∼13 µrad, then rapidly broadened at Δθ > 13 µrad. The minimum width achieved was 400 meV FWHM. Assuming the autocorrelation width of reference spectra (crosses) of 200 meV FWHM reflects the true spike width, a deconvolved autocorrelation width of 350 meV FWHM was obtained on a Gaussian assumption, giving an estimate on the energy resolution of ∼250 meV. This result indicates that the energy resolution was successfully improved by a factor of approximately two compared with a single flat-crystal analyzer.

However, note that the energy resolution achieved in this experiment was lower than expected (dashed line in Fig. 3 ▸). This discrepancy may be attributed to the finite penetration depth within the analyzer crystals. The incident X-ray beam penetrates the crystal surface while being diffracted continuously. In the special case of an incident beam with zero divergence, bandwidth and transverse size, this process causes spatial – rather than angular – blurring along the dispersion direction of the incident beam, resulting in a finite transverse size of the reflected beam. This spatial blurring, originating from the analyzer crystals, corresponds to *p*_ana_. Consequently, the widths of the PSF and the resolution function broaden, as described by equation (1). The impact of penetration depth is discussed quantitatively below.

## 4. Discussion

This section focuses on the impact of penetration within the analyzer crystals. The penetration depth *z_e_* is defined as the distance from the crystal surface at which the attenuation factor equals 1/*e*. This can be expressed as (Authier, 2001 ▸)
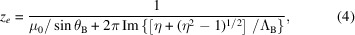
where μ_0_ is the linear absorption coefficient in the forward direction, Λ_B_ is the extinction distance and η is the deviation parameter. Here, we assumed the symmetric case, as is the case with the MCA used in this experiment. With the angular deviation from the exact Bragg angle δθ and a complex constant δ_B_ (where the real part corresponds to half of the intrinsic Darwin width), η can be rewritten as
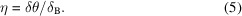
The penetration depth *z*_*e*_ is approximately 1 µm near the exact Bragg angle (δθ = 0) for Si 220 diffraction at *E* = 10.4 keV, increasing to tens of micrometres or more at a large η. We assumed that the beam profile of diffracted X-rays can be expressed as the convolution of the incident profile and an exponential decay function with a coefficient of 2*z*_*e*_cosθ_B_ directed toward the low-angle side. This spread in the spatial profile results in a finite *p*_ana_, which leads to worsened energy resolution in accordance with equation (1). Note that the PSF of the MCA varies with angle due to different η. Considering a pair of two double-bounce reflections with an angular offset Δθ in the MCA, we obtain the PSF of the MCA, *P*_MCA_, as
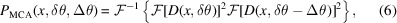
where 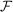 and 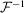 denote the Fourier and inverse Fourier transformations, respectively. The exponential decay function *D*(*x*, δθ) is expressed as
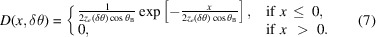
Using equations (4)–(7), the PSF of the analyzer was calculated for each angle and converted into an angular spread function (ASF) by dividing by *L* (*i.e.* with α = *x*/*L*). The ASF was then convolved with the angular resolution function of the MCA, *R*_MCA_(δθ, Δθ). The resultant angular resolution function can be written as
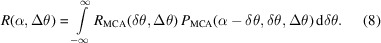
Finally, the autocorrelation width of *R*(α, Δθ) was evaluated, taking the spike width into account. The calculated autocorrelation width showed good agreement with the measured width at Δθ 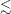
 16 µrad, as shown in Fig. 3 ▸ (solid line). Therefore, it is reasonable to conclude that spatial broadening due to penetration within the crystals is a dominant factor in deteriorating the energy resolution.

**Figure 4:**
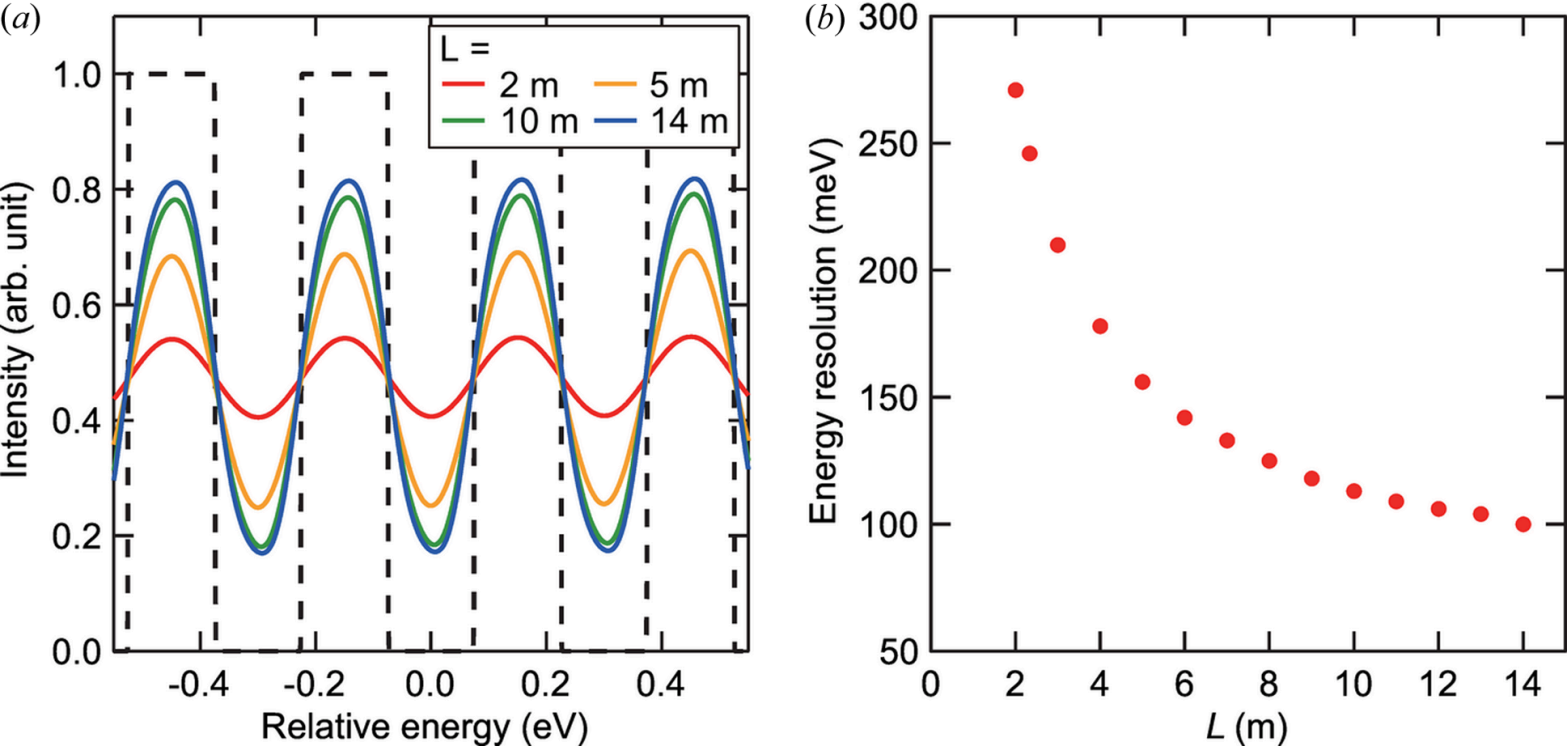
(*a*) Simulated output spectra (solid lines) at *L* = 2, 5, 10 and 14 m for a comb-like input spectrum with a comb width of 150 meV (dashed line). The visibility of the simulated spectra becomes higher at larger *L*. (*b*) Calculated highest energy resolution as a function of *L*.

A straightforward way for improving the energy resolution is to increase the detector distance, *L*. To quantify the energy resolution independently on the shape of the angular resolution function, output spectra were simulated for a comb-like input spectrum with a duty ratio of 0.5 [see Fig. 4 ▸(*a*)]. We defined the energy resolution as the comb width that can be resolved with a visibility of 0.5. Fig. 4 ▸(*b*) shows the calculated energy resolution as a function of *L*. At *L* = 2.334 m (matching the experimental setup), the highest energy resolution was estimated to be 246 meV at Δθ = 12.6 µrad, which is consistent with the experimental observations. A high energy resolution of 113 meV could be achieved at *L* = 10 m, reaching 100 meV at *L* = 14 m that is approximately one fifth of the single-analyzer resolution.

Another approach is the use of a parabolic focusing lens or mirror. At the back-focal plane of such a focusing device, only the angular profile of the incidence beam is observed. Therefore, placing a focusing device downstream of an MCA and locating a 2D detector at the back-focal plane allows the collection of resolution-enhanced spectra at a reasonable *L* without the impact of spatial broadening. A potential challenge for this approach is developing high-precision focusing devices with apertures of several millimetres.

A practical alternative involves the combination of a highly divergent beam with a detuned MCA operating in middle-order diffraction. A focusing mirror that produces a divergent beam with Ω ≃ 5 mrad can be designed while satisfying total-reflection conditions at photon energies around 10 keV. At Ω = 5 mrad, a broad photon energy range of >80 eV can be achieved using Si 311 or 400 diffractions, with intrinsic energy resolutions estimated at ∼270 meV and ∼240 meV, respectively, at 10 keV. The detuned MCA approach demonstrated in this study should enhance the energy resolution to ∼100 meV or less, even at *L* ≃ 3 m.

## 5. Summary

A simple method for improving the energy resolution of the dispersive single-shot X-ray spectrometer was proposed and demonstrated at BL3 of SACLA for 10.4 keV SASE XFEL pulses. The detuned MCA, consisting of two Si(220) channel-cut crystals arranged in the non-dispersive geometry with an angular offset, enhances the energy resolution by a factor of two compared with a single analyzer. Further improvement in energy resolution should be achieved by increasing the detector distance, which would suppress the influence of X-ray penetration inside the analyzer crystals. Additionally, it was observed that Fresnel diffraction from the finite aperture of the focusing mirror created artifacts in the measured spectra, limiting the effective energy range to approximately half of the total range. The use of a large focusing mirror with an aperture greater than the beam size, along with a long detector distance of over 14 m, should enable full spectral characterization of broadband XFEL pulses, achieving an energy resolution of ∼100 meV and an energy range of ∼80 eV without considerable limitations in operational photon energy. Detailed spectral information obtained through the proposed scheme should allow for the reconstruction of the complex spectral/temporal profiles of XFEL pulses, on a single-shot basis along with electron bunch profiles measured by a transverse deflecting cavity (Christie *et al.*, 2020 ▸) via statistical approaches (Robles *et al.*, 2023 ▸) and spectral phase interferometry for direct electric-field reconstruction (De Ninno *et al.*, 2015 ▸; Fuji *et al.*, 2023 ▸), as well as advanced X-ray spectroscopy techniques, *e.g.*X-ray ghost spectroscopy (Klein *et al.*, 2023 ▸).
